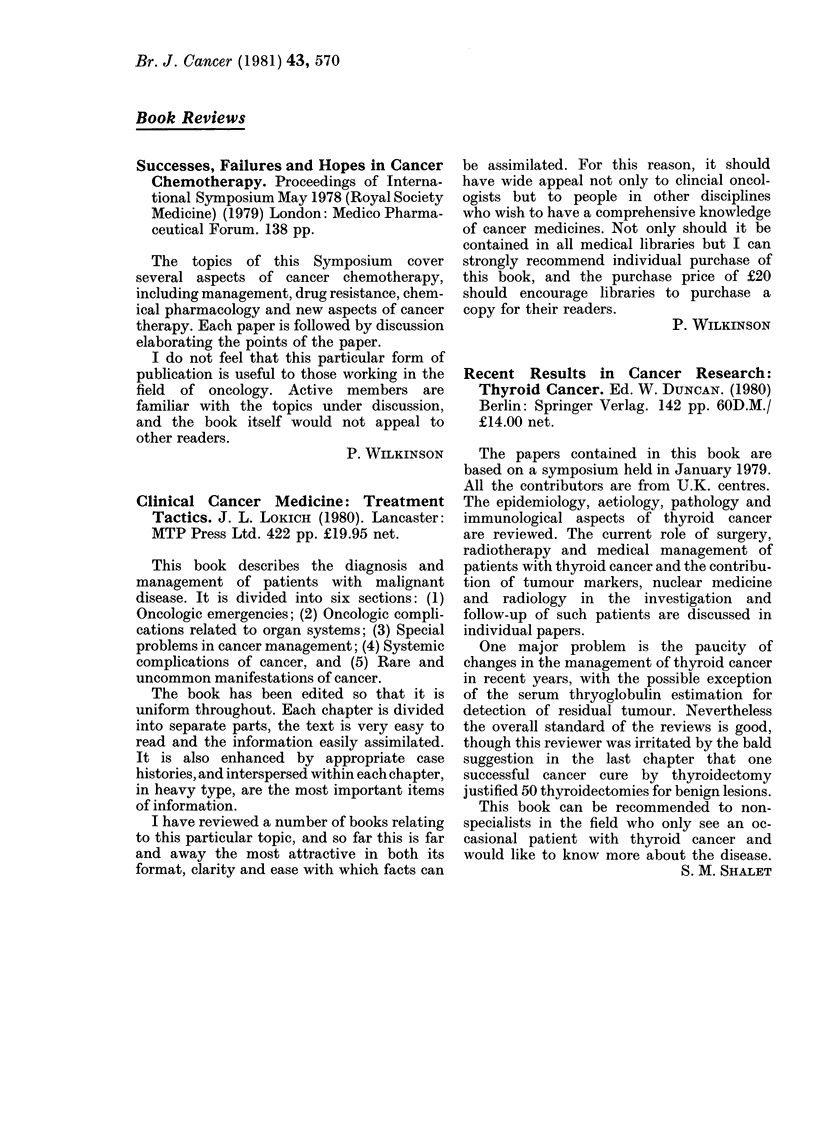# Successes, Failures and Hopes in Cancer Chemotherapy

**Published:** 1981-04

**Authors:** P. Wilkinson


### *Book Reviews*

**Successes, Failures and Hopes in Cancer Chemotherapy.** Proceedings of International Symposium May 1978 (Royal Society Medicine) (1979) London: Medico Pharmaceutical Forum. 138 pp.

The topics of this Symposium cover several aspects of cancer chemotherapy, including management, drug resistance, chemical pharmacology and new aspects of cancer therapy. Each paper is followed by discussion elaborating the points of the paper.

I do not feel that this particular form of publication is useful to those working in the field of oncology. Active members are familiar with the topics under discussion, and the book itself would not appeal to other readers.

P. WILKINSON